# Behavioral economics: who are the investors with the most sustainable stock happiness, and why? Low aspiration, external control, and country domicile may save your lives—monetary wisdom

**DOI:** 10.1007/s13520-022-00156-z

**Published:** 2022-11-16

**Authors:** Ningyu Tang, Zhen Li, Jingqiu Chen, Thomas Li-Ping Tang

**Affiliations:** 1 Department of Organization Management, Antai College of Economics and Management, Shanghai Jiao Tong University, Shanghai, 200030 China; 2 Management School, Hainan University, Haikou, Hainan 570228 China; 3 College of Business, Texas Woman’s University, Denton, TX 76204 USA; 4 Department of Management, Jennings A. Jones College of Business, Middle Tennessee State University, Murfreesboro, TN 37132 USA

**Keywords:** Stock volatility, Boom-bust, Bull-bear markets, SSE, Shanghai Stock Exchange Index, Greed, Avaricious monetary aspiration, Love of money, Internal vs. external-locus-of-control, Domicile, Residence, City vs. country, Identity, Environmental capital, HOPE, Portfolio changes, Utility, Stock happiness, SWB, Serenity, Behavioral economics, Finance, Prospect theory, Gains vs. losses, Probability, Risk seeking vs. risk aversion, Health, Stress, Mindfulness, Values, Self-transcendence vs. self-enhancement, Sacred vs. secular, TPB, Attitude, Control, Norms, Behavior, China

## Abstract

Slight absolute changes in the Shanghai Stock Exchange Index (SHSE) corresponded to the ***city’s*** immediate increases in coronary heart disease deaths and stroke deaths. Significant fluctuations in the Shenzhen Stock Exchange Index (SZSE) corresponded to the ***country’s*** minor, delayed death rates. Investors deal with money, greed, stock volatility, and risky decision-making. Happy people live longer and better. We ask the following question: Who are the investors with the highest and most sustainable stock happiness, and why? Monetary wisdom asserts: Investors apply their deep-rooted values (avaricious love-of-money aspiration and locus of control, Level 2) as a lens to frame critical concerns in the proximal-immediate (Shanghai Stock Exchange Index changes, Level 1) and the omnibus-distal contexts (domicile: city vs. country, Level 2) to maximize expected utility (portfolio changes, Level 1) and ultimate serenity (stock happiness, Level 1). We collected ***multilevel*** data—the ***longitudinal*** SHSE and 227 private investors’ ***daily*** stock happiness and portfolio changes for 36 consecutive trading days in four regions of China. Investors had an average liquid asset of $76,747.41 and $54,660.85 in stocks. This study is not a “one-shot” game with “nothing at stake.” We classified Shanghai and Beijing as the city and Shenzhen and Chongqing as the country. Our cross-level 3-D ***visualization*** reveals that regardless of SHSE volatility, investors with low aspiration, external control, and country domicile enjoy the highest and most sustainable stock happiness with minimum fluctuations. Independently, investors with low aspiration, external control, and country domicile tend to make *fewer* portfolio changes than their counterparts. Behaviorally, less is more, debunking the myth—risky decisions excite stock happiness. Our longitudinal study expands prospect theory, incorporates attitude toward money, and makes robust contributions to behavioral economics and business ethics. We help investors and ordinary citizens make happy, healthy, and wealthy decisions. Most importantly, the life you save may be your own.

Many Nobel Laureates in Economic Sciences have studied the stock markets, decision-making, human judgment, investment, and behavioral economics. We highlight several Nobel Prize winners. Their seminal works inspire our present study. Regarded as the father of modern finance, Eugene F. Fama (1970, 1998) demonstrated difficulties predicting stock prices in the short term. On the other hand, Robert J. Shiller (2015) predicted the 2008 housing crash in the long run. Interestingly, Fama and Shiller won the 2013 Nobel Prize (with Lars Peter Hansen), taking the disagreement to a new level.

On October 10, 2022, the Royal Swedish Academy of Sciences in Stockholm announced that three economists shared the Nobel Prize in economic sciences for their research on banks and financial crises. These three American economists, Former U.S. Federal Reserve Chair Ben S. Bernanke, The Brookings Institution, Douglas W. Diamond, University of Chicago, and Phillip H. Dybvig, Washington University, made vital contributions to the 2008–2009 financial crisis, illustrating the role banks played in this profound event. “Avoiding bank collapses is vital” to people, their savings, money, investment, long-term loans to borrowers, and the banks in the USA and around the world. In the present study, we investigate stock volatility’s impacts on investors’ stock happiness and portfolio changes and identify investors with sustainable stock happiness. Our study makes critical contributions to the behavioral economics and behavioral finance literature, demonstrating how investors and ordinary citizens can make “vital” happy, healthy, and wealthy financial decisions at the individual level.

Best known for his work in applying psychological insights to economic theory and creating the field of behavioral economics Daniel Kahneman and his colleague Amos Tversky developed the prospect theory. The prospect theory explores decision-making under uncertainty and frames decisions in the gains-losses domain and high-low probability (Kahneman & Tversky, 1984; Tversky & Kahneman, 1970). Kahneman also advised us “There may also be cultural differences in the attitude toward money” when explaining the endowment effect (2011, p. 298). We incorporate investors’ attitude toward money and locus of control to explore the impacts of stock volatility in the domain of gains (bull markets) and losses (bear markets) on investors’ objective decision-making and subjective stock happiness for 36 consecutive trading days in China.

George A. Akerlof and Rachel E. Kranton stated that individuals’ social identities, “a person’s sense of self,” are essential in decision-making (2000, p. 715). Since the omnibus context impacts investors’ attitudes, health, and behavioral intentions, we include investors’ place of residence (city vs. country) to investigate their stock happiness in four regions of China.

Kahneman and Deaton (2010) explored income, evaluation of life, and emotional well-being. High income (beyond $75,000) improves individuals’ evaluation of life but *not* emotional well-being. The rich’s material possessions, new cars, and big houses do not make them happy. Thaler (2015) nudged individuals to make healthy, happy, and wealthy decisions. Our study is the first longitudinal research exploring the impacts of Chinese investors’ avaricious monetary aspiration (greed or love of money), locus of control, domicile (city vs. country), and longitudinal stock volatility on investors’ daily subjective stock happiness and objective portfolio changes for 36 consecutive trading days across four regions of China. We provide our rationale below.

This research focuses on Chinese stock investors. We offer a brief history here. Chinese Communist Party closed Shanghai Stock Exchange in 1949. After the economic reform, China reopened it in 1990. As of June 2022, Shanghai Stock Exchange is the third-largest in the world (behind the NYSE and NASDAQ). Shiller successfully predicted the 2008 housing crash in the USA yet could not prevent the financial crisis from impacting people in different parts of the world (Shabri Abd Majid & Hj Kassim, 2009; Tang & Ibrahim, 1998; Tang & West, 1997; Tang et al., 2002). During the financial crisis, SHSE Index reached an all-time high (6092.06) in China’s emerging markets on October 16, 2007. It dropped to its lowest point (1706.70) on November 4, 2008. The absence of exposure to stock investment for four decades may reduce individual agility to respond appropriately. What were the reactions to this financial crisis in China?

As expected, the SHSE Index volatility was associated with Chinese investors’ low index happiness (91.4%), low stock happiness (72.4%), high psychological stress (71.8%), and low happiness overall (65.4%) (China Investor Happiness Survey, 2008). Furthermore, researchers examined the Shanghai Stock Exchange Index (SHSE,上海证券交易指数) changes and the Shanghai Center for Disease Control and Prevention’s (CDC) death rates (2006–2008) and revealed shocking discoveries. Their “1-day lag model” of each 100-point absolute change of the SHSE Index corresponded to a 5.17% increase in *coronary heart disease (CHD) deaths* (Ma et al., 2011) and a 3.22% increase in *stroke deaths* in Shanghai (上海) (Zhang et al., 2013). In Southern China, a “15–25-day lag model” of each 800-point change of the Shenzhen Stock Exchange Index (SZSE, 深圳证券交易指数, the fifth largest in the world) and the Guangdong Province CDC’s death rates showed increases in *cardiovascular mortality rates* in Guangzhou (广州, 2.38% in the boom cycle vs. 2.08% in the bust cycle) and Taishan (台山, 2.08% in the bull market vs. 1.65% in the bear market) (Lin et al., 2013). Please note that the CDC in Shanghai and Guangdong Province reported the death rates for the population in general but *not* for investors.

However, in the USA, the stock market crash in October 2008 showed no impact on Los Angeles’s death rates (Schwartz et al., 2012). The NYSE volatility did not disturb the suicide rates in New York City (1999–2006) (Nandi et al., 2012). We attempt to identify the reasons for the differences in these events.

Consumers fall prey to the heuristics—buy past winners and sell past losers (Johnson et al., 2005). The media effect is more potent in a *bull* market than in a bear market (Huang, 2019) and in a *local* market than in a foreign market (Li et al., 2021). These discoveries help explain the relationships between country investors’ risk-seeking behaviors and the high mortality rate in the bull market. We summarize our observations and contributions below.

First, changes in the Shanghai Stock Exchange Index (SHSE) and death rates were more robust than in the Shenzhen Stock Exchange Index (SZSE) and mortality rates. Hence, we select the Shanghai Stock Exchange Index (SHSE) for this research. Second, the stock index had a more substantial impact on mortality rates in the *city* (Shanghai/上海) than in the *country* (Guangzhou/广州 and Taishan/台山). Index changes impacted mortality rates in China but not in the USA. The importance of contextualization (Johns, 2017; Rousseau & Fried, 2001) motivates us to incorporate investor domicile (city vs. country) in exploring investor stock happiness.

Third, happy people live longer and better (Gan, 2020). Happy cities have lower suicide rates (Park & Peterson, 2014). Jewish concentration camp survivors suffered from stress and were twice more likely to die of cancer, CHD, and other causes than those in the control group without stress (Grossarth-Maticek et al., 1994). These findings suggest that people with sustainable longitudinal stock happiness will live longer and better and are less likely to suffer from stress and death. The omnibus context matters (Al Halbusi et al., 2022).

Fourth, Nobel Laureate Richard H. Thaler (2015) stated that prospect theory’s experiments involve a “one-shot” game (2015, p. 49). Participants typically have “nothing at stake.” “For economists that meant they could be safely ignored” (p. 47). “People think about life in terms of changes, not levels.” Changes “make us happy or miserable” (p. 31). Fifth, N. Tang et al. (2018) selected 229 investors (MBA students in Shanghai) and examined the love of money and longitudinal data across 30 consecutive trading days during the financial crisis. These findings and inspirations motivate us to investigate ordinary investors.

Our present study makes the following contributions. We robustly advance the existing literature by exploring the “longitudinal” ***changes*** of the SHSE Index and investors’ daily responses in stock happiness and portfolio changes for 36 consecutive trading days. We recruited ordinary investors from an investment management company. Investors provided avaricious aspirations, external locus of control, and domicile in four regions of China. We collected longitudinal (public and private) data and conducted our study at a different time. Investors had an average liquid asset of $76,747.41 and invested $54,660.85 in stocks. We frame our constructs in the prospect theory’s theoretical framework. Our longitudinal study of stock happiness and portfolio changes is not a “one-shot” game with “nothing at stake” (Thaler, 2015). We challenge the myth: “Greedy investors” with an “*internal* locus of control” living in “large megacities” will achieve sustainable stock happiness in an “*uncontrollable*” stock market. Our ***cross-level*** discoveries provide novel three-dimensional (3-D) visualization. Investors with low aspiration, external control, and country domicile enjoy the highest and most sustainable stock happiness. Independently, investors with low aspiration, external control, and country domicile tend to make *fewer* portfolio changes than their counterparts. Behaviorally, less is more, debunking the myth. We make robust theoretical contributions to behavioral economics, business ethics, stress, health, and well-being. Our practical implications help investors and ordinary citizens make happy, healthy, and wealthy decisions. Essentially, the life you save may be your own (Schelling, 1985).

## Theory and hypotheses

Following monetary wisdom, we present our overarching theory with constructs (measured variables) as follows: Decision-makers (private investors) select their deep-rooted personal values (avaricious monetary aspiration/love of money and locus of control) as a lens and “frame” the critical concerns in the immediate (daily stock index volatility) and the omnibus (investor domicile-residence: city vs. country) contexts to maximize their expected utility (investment-portfolio changes) and ultimate serenity (stock happiness) across people, context, and time (Tang, 2021; Tang et al., 2018a, b, 2022).^1^ Monetary aspiration, locus of control, and domicile (city vs. country) are the individual-level variables (Level 2). We explore the impact of the longitudinal objective SHSE Index changes (Level 1 independent variables) on investors’ daily subjective index happiness, stock happiness, and objective behaviors—portfolio changes (Level 1 dependent variables). Our 227 individual investors (Level 2) provide all these repeated within-subjects measures (Level 1). Thus, investors’ Level 2 variables robustly impact Level 1 dependent variables. Our data allow us to conduct cross-level analysis and offer three-dimensional visualization.

**Fig. 1:**
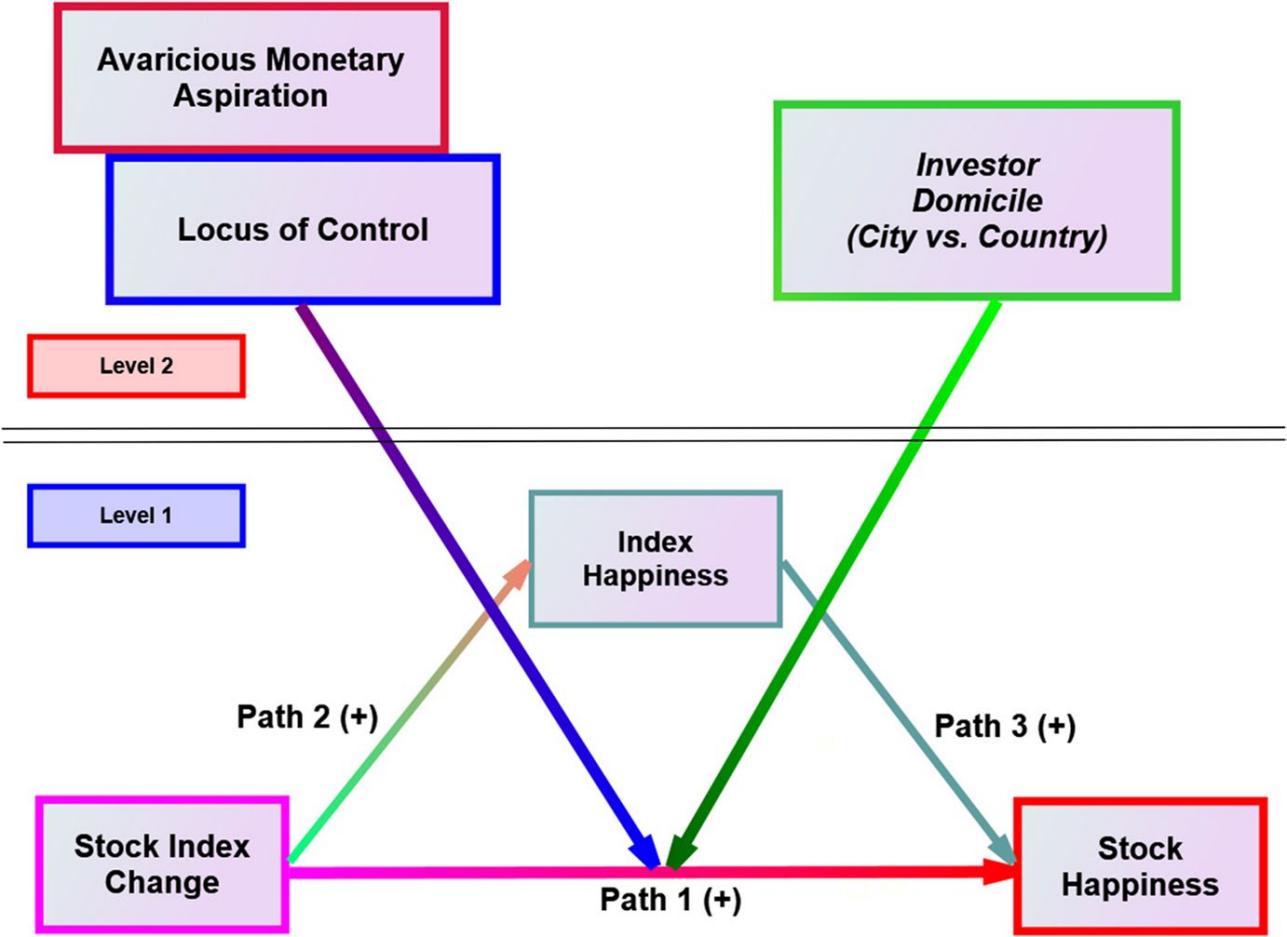
The theoretical model of aspiration, control, and domicile (Level 2) on the relationships between longitudinal stock index changes and longitudinal stock happiness (Level 1)

The theory of planned behavior (TPB) (Ajzen, 1991) suggests that attitude, control, and norms predict behavioral intention, which predicts actual behavior (Gopi & Ramayah, 2007; Kirchler et al., 2008; Tang & Baumeister, 1984). Tang (1992, 1993) followed the ABC (Affective-Behavioral-Cognitive) model of attitudes and developed the Money Ethic Scale (MES). Decision-makers use the meaning of money as their “frame of reference” to examine their everyday lives (1992, p. 201). Following TPB, we theorize that investors’ love of money attitudes, locus of control, social norms (domicile: city vs. country), and longitudinal SHSE Index changes jointly ***predict*** their daily index happiness, stock happiness, and actual portfolio changes (behaviors) for 36 consecutive trading days. Figure 1 illustrates our theoretical model. We introduce significant constructs below.

## Avaricious monetary aspiration (the love of money attitude)

### Money

For centuries, the clashes of conflicting values—self-transcendence (sacred values) vs. self-enhancement (secular values)—have caused many conflicts in our lives (Grouzet et al., 2005; Schwartz, 1992). In economics, Adam Smith stated: Money is an instrument of commerce and a measure of value. In psychology, Harvard Psychologist David McClelland proclaimed: The meaning of money is “in the eye of the beholder” (McClelland, 1967, p. 10; Tang, 1992). Globally, money is the only universal language that everyone understands without speaking. Money is a tool and a drug (Lea & Webley, 2006). As a tool, money satisfies people’s basic physiological and psychological needs. In emerging markets, Chinese people are unhappy (Easterlin, 2012) because they mostly compare themselves with the rich. When people treat money as a drug, the more money they have, the more they want. Keeping up with the Joneses (Luna-Arocas & Tang, 2015) and comparing with the rich will cause the “fear of missing out” (FOMO) (Good & Hyman, 2020) and the hedonic treadmill (Brickman & Campbell, 1971; Gentina & Tang, 2022). Even if they gain more possessions, they return to the same low level of happiness. However, happiness does not depend on what you have or who you are; it solely relies on what you think (Carnegie, 1936).

Baumeister et al. (2013) suggested that there are differences between a happy life (a current orientation, a taker) and a meaningful life (the integration of past, present, and future, a giver). Happiness is ***absolute*** in consumption but ***relative*** in the context of money (Hsee et al., 2009). In our modern societies, time is money. Decision-makers process money ***analytically*** and time ***affectively*** (Lee et al., 2015). Nobel Laureate Daniel Kahneman stated “There may also be cultural differences in the attitude toward money” when explaining the endowment effect (2011, p. 298). We answer Kahneman’s call, follow his advice, and incorporate “attitude toward money” in studying decision-makers’ stock happiness and portfolio changes.

### Money ethic scale and the love of money scale

Following the ABC model, Tang developed the Money Ethic Scale (MES) (1992, 1993) and explored the meaning of money. Tang and Chiu (2003) expanded the MES and coined the love of money construct. The avaricious monetary aspiration construct consists of Factors Rich, Motivator, and Important (Tang & Chiu, 2003; Tang et al., 2018a, b). Avaricious investors act proactively, take risks, and make profits. Monetary aspirations impact goal-setting and decision-making (Howard et al., 2015; Tang & Sarsfield-Baldwin, 1991).

Factor Rich, the ***affective*** component, deals with money’s love or hate emotions and predicts the magnitude of cheating in experiments (Chen et al., 2014) because people love to be rich (Harpaz, 1990). Factor Motivator, the ***behavioral ***component, measures people’s behavioral intentions and predicts the cheating percentages (actions) in laboratory experiments. Money is a Motivator. Pay-for-performance programs influence behavior and are superior to other approaches to improving actual performance (Locke et al., 1980). Factor Important, the ***cognitive ***component, explores the importance of money. Males and females ranked pay fifth and seventh in importance for “themselves.” Interestingly, men and women rated pay as the most crucial goal for “others” (Jurgensen, 1978). We summarize four decades of research in the following paragraphs:

Mitchell and Mickel (1999, p. 571) suggested that the Money Ethic Scale is one of the most “well-developed” and “systematically” used measures of money attitude in the literature. Observing Euro banknotes increase from €5 to €500, greedy individuals express their emotional arousals logarithmically, illustrating money’s rewarding properties (Giuliani et al., 2021; Manippa et al., 2021). The avaricious love-of-money attitude is related to high risk-taking actions in an ERP study (Jia et al., 2013) and high risk-tolerance intention (Tang et al., 2008). Saving money buffers death anxiety (Zaleskiewicz et al., 2013). “The love of money results in objectification” (Wang & Krumhuber, 2017, p. 354). Money attitude impacts pay dissatisfaction (Luna-Arocas & Tang, 2015), pay differential disparity (Tang, 1996; Tang et al. 2000a), dishonest intentions (Gentina & Tang, 2018; Sardžoska & Tang, 2015; Tang et al., 2022), and short-term and long-term investment decisions (Chaudary et al., 2022). A high love-of-money score reveals one’s favorable attitude toward money. People in positive (negative) moods tend to make optimistic (pessimistic) judgments (Grevenbrock, 2020; Johnson & Tversky, 1983).

The love-of-money attitude predicts unethical behavioral intentions in panel studies (Tang & Chen, 2008; Tang & Tang, 2010), cheating behaviors in experiments (Chen et al., 2014), low course grades in a business course (Tang, 2016), low stock happiness (Tang et al., 2018), and voluntary turnover 1.5 years later (Tang et al., 2000b). The love of money creates strong emotional reactions and helps them maximize utility for their financial gains (Tang & Gilbert, 1995).

Scholars have substantiated monetary wisdom—the relationships between the love-of-money construct and positive and negative outcomes—in more than 50 countries across six continents (Tang, 2020, 2021), including under-researched regions.^2^ Researchers have cited this money-related construct in numerous textbooks on compensation (Gerhart, 2023), human resource management (Phillips, 2022), management (Bateman & Snell, 2013), organizational behavior (Colquitt et al., 2021), and the psychology of money (Furnham, 2014).

In a 20-country study involving 3600 investors, Chinese investors’ love of money ranked second behind India, whereas the USA ranked sixth and the Netherlands ranked 20^th^ (Authers, 2016; Bloomberg, 2016). High investor love of money is bad for investors’ financial health. The opportunity to get rich quickly in China’s *emerging markets* exists. Thinking about money (Vohs et al., 2006) prompts them to pay attention to the SHSE Index and take risks, leading to high stress. A recent study in Pakistan’s emerging markets showed that the relationship between the love of money attitude and short-term investment decisions is much stronger for investors with lower incomes than those with higher incomes. The relationships between the love of money attitude and short-term and long-term investment decisions are much more vital for investors without future inheritance expectations than those with future inheritance expectations. Interestingly, with future inheritance expectations, investors have a higher ***magnitude*** (level) of short- and long-term investment decisions than those without future inheritance expectations. Thus, the have-nots (investors with low income and without inheritance expectations) demonstrate a higher ***intensity*** between investors’ love of money and investment decisions than the haves. These findings support the Matthew Effect in investment decisions in emerging markets (Chaudary et al., 2022). China is also an emerging market. Chinese investors may behave similarly.

However, investors in *developed* economies “have little interest in speculation and are long-term investors by nature” (Clark-Murphy & Soutar, 2004, p. 539). Investors with well-diversified portfolios do not spend much time or money managing their investments, yet they gain considerable enjoyment and personal satisfaction. These findings explain the differences in stock volatility and mortality between China and the USA.

In the present study, we follow the theory of planned behavior (TPB) and employ money attitude to predict longitudinal stock happiness and investment portfolio changes. Ceteris paribus, high (low) avaricious monetary aspiration leads to low (high) stock happiness and high (low) stock portfolio changes (Tang et al., 2018a, b).

## Internal–external locus of control

Research on stock volatility and death rates reminds us of a classic study in the literature. Brady (1958) trained executive monkeys to push a button every 20 seconds to avoid electric shocks. With a continuous “6-hour on and 6-hour off” schedule, executive monkeys died from a perforated ulcer 23 days later due to stress (not electric shocks). Executive monkeys’ attempts to avoid and control the electric shocks led to dire consequences. In the control group, the yoked monkeys took no action and survived. We argue that internal locus of control may be detrimental to investor happiness.

Rotter’s (1966) Internal–External Locus of Control Scale (I-E) assesses how people attribute the cause of events to themselves or the external environment. High internal locus-of-control individuals have high task performance, income, satisfaction (Spector, 1982), reservation wages, and a high probability of reemployment (Caliendo et al., 2015; Judge & Bono, 2001; Lim et al., 2003). Work locus of control corresponds to well-being at work (Spector et al., 2002). Asians exercise less control than Americans (Spector et al., 2004). Police officers’ hardiness (control, challenge, and commitment) moderates the relationships between police stress (Time 1) and absenteeism (Time 2, six months later, Tang & Hammontree, 1992). Many people have the following shared beliefs: Internal locus-of-control investors attempt to control their investments, take challenging actions quickly, make risky and panicky decisions, and enjoy stock happiness in the bull market to achieve expected utility aspiration and emotional exhilaration.

We challenge this myth for the following reasons: First, economists have difficulties predicting short-run stock volatility (Fama, 1998). Second, individual private investors have little control over the stock index changes. Third, when internal-locus-of-control investors attempt to exploit the uncontrollable SHSE Index, “the resulting psychological conflict can bring negative attitudinal or behavioral outcomes” (Ng et al., 2006, p. 1,074). Finally, please recall the robust empirical relationships between changes in SZSE and country residents’ high mortality rate in the *bull* market. High internal locus-of-control investors may experience increased stress and low stock happiness. Thus, we theorize that the internal–external locus of control modifies the relationship between the love of money and stock happiness (Li et al., 2020; Lu et al., 2000). On the other hand, external-locus-of-control investors may have little interest in speculation. They are long-term investors, relinquish their control of the stock markets, make fewer changes in their portfolio, and enjoy higher stock happiness-serenity than their internal locus-of-control counterparts.

## Investor domicile (city vs. country)

George A. Akerlof won the 2001 Nobel Prize in Economic Sciences. Akerlof and Kranton (2000) demonstrated how identity could affect individual interactions and substantively change “conclusions of previous economic analysis” (p. 715). Proshansky (1978) coined *place identity*—the social and cultural processes involved in developing self-identity. Proshansky defined it as a substructure of self-identity consisting of memories, ideas, feelings, attitudes, values, preferences, meanings, and conceptions of behavior and experience that occur in places that satisfy an individual’s biological, psychological, social, and cultural needs (Proshansky et al., 1983). Identification with the place provides many benefits—helping residents gain a better quality of life (Harris et al., 1995), physical and psychological health, satisfaction with social relationships, and physical environment (Tartaglia, 2012).

“Home is where the heart is.” Anton and Lawrence (2014) found that rural residents reported higher place identity than urban dwellers. We visualize stock happiness through the lens of investors’ domicile (city vs. country). We theorize that investor domicile shapes their thinking, feelings, and behavior (Oishi, 2015) and helps us understand person-environment interactions (Treviño, 1986). Happiness is relative in the context (Hsee et al., 2009). We selectively reviewed several critical research findings below.

Among 56,000 Londoners, 216 communities vary in life satisfaction and personality patterns (Jokela et al., 2014; Oishi, 2015). Cultural tightness coexists with urbanization, economic growth, and happiness over time among 11,662 individuals across 31 provinces in China (Chua et al., 2019). Higher demand for water and labor causes Southern rice growers in China to have a higher level of *holistic thinking* than Northern wheat growers (Chen et al., 2022a; Talhelm et al., 2014). Ambient temperature is associated with human personality (Wei et al., 2017) and high-risk financial decisions (Huang et al., 2014).

Firms in regions with high happiness have increased R&D intensity and firm investment (Chuluun & Graham, 2016). Male residents in Watts (the Nickerson Gardens public housing project) have earnings of only $7000 a year and a 45% chance of being incarcerated on any given day. The neighborhood could be an *engine* for success or a *brake* on their ambitions (Chetty et al., 2014).^3^ The conservation of resources (COR) theory suggests that resource surpluses help individuals reduce stress and experience euphoria (Hobfoll, 1989). High self-esteem people have low behavioral plasticity (Brockner, 1988; Tang & Reynolds, 1993).

In materialistic societies, ***time is money***. Consumers process money ***analytically*** and time ***affectively*** (Lee et al., 2015). Modern technologies help individuals perform their tasks faster and better (Gentina et al., 2018a, b). People reported greater happiness spending money on a time-saving purchase than on a material purchase (Whilans et al., 2017). Placing a *price* on “time” impairs our ability to enjoy pleasurable experiences. People become too ***impatient*** to smell the roses (DeVoe & House, 2012). In the USA, living in the Big Apple (New York City) differs from living in the South. People in New York City mind their own business and are incredibly more fast-paced than those in the South. In the country, people always ask how you are doing and are concerned about other peoples’ lives (Lyles, 2015).

In a study of 10 European ***city-forest*** comparisons, city birds sing their urban songs shorter, faster, and with higher minimum frequency than their country counterparts (Slabbekoorn & den Boer-Visser, 2006), supporting Aesop’s fable of the *city* mouse and *country* mouse. City noise and air pollution reduce people’s subjective well-being, SWB (Diener et al., 2018; Zheng et al., 2019). Healthy environments and green spaces improve SWB (Diener et al., 2017, 2018; Wicks et al., 2022). Our domicile shapes our social norms, identities, sense of self, SWB, and behavioral tendencies (Akerlof & Kranton, 2000), impacting investors’ happiness and investment decisions. We now turn to investors’ domicile.

### City vs. country

In the present study, we classified Shanghai and Beijing as the city and Shenzhen and Chongqing as the country using the following objective and subjective criteria. First, most importantly, the Shanghai Stock Exchange’s 100-point change was associated with ***immediate*** (one-day) and much ***more robust*** impacts on CHD and stroke mortality rates in the ***city*** (Shanghai, the financial capital) (Ma et al., 2011; Zhang et al., 2013); whereas the Shenzhen Stock Exchange’s 800-point change was associated with ***delayed*** (15–25-Day) and ***weaker*** impacts on CHD mortality rates in the ***country*** (Guangzhou and Taishan) (Lin et al., 2013). Shenzhen, in the South, is geographically close to Guangzhou (population 14.5 million, distance 105 km) and Taishan (population less than 1 million, distance 136 km).

Second, a higher GDP growth rate in the environmental context predicts individual happiness in China (Fu, 2018). Shanghai is the ***financial capital*** of China, with a GDP of $810 Billion, equivalent to the Netherlands’ GDP. Shanghai Stock Exchange (SHSE) is the ***third largest*** stock exchange. Beijing is the ***political capital*** with a GDP of $664 Billion, equivalent to Switzerland’s GDP. Shenzhen’s GDP was $491 Billion, like Sweden’s GDP. Chongqing’s GDP reached $425 Billion, close to Thailand’s GDP. The GDPs in Shanghai and Beijing (the developed economy) are robustly higher than in Shenzhen and Chongqing (the developing economy).

Third, the most popular real estate quote of all time is “Location, location, location.” This principle applies to our research’s contextualization (Johns, 2017; Rousseau & Fried, 2001). For contextualization, we turn to ***location***. However, it is not easy to compare apples with oranges. **Chongqing** (population 32.05 million, area 82,400 km^2^) is located remotely in a central southwest location—***far away*** from China’s historical and epic centers on the east coast—**Shanghai** (population 24.87 million, area 6340 km^2^, distance 1440 km/895 miles) and **Beijing** (population 21.89 million, area 16,410 km^2^, distance 1458 km/906 miles). **Shenzhen** (population 17.58 million, area 2000 km^2^) in Southern China is 1435.5 km/897 miles south of Shanghai. Shanghai and Beijing have larger populations than Shenzhen. Chongqing has the largest population (32.05 million) but is primarily rural due to its largest area (82,400 km^2^), compared to Shanghai (6340 km^2^) and Beijing (16,410 km^2^).

Shanghai’s SHSE is the third largest stock exchange in the world, much more substantial and decisive than SZSE’s fifth ranking. Following Akerlof and Kranton’s (2000) ***social identity***, we conclude that Shanghai is the financial capital and Beijing is the political capital of China, but Shenzhen and Chongqing are ***not***.

Moreover, city investors have higher exposures to money, the SHSE Index, time pressure (DeVoe & Pfeffer, 2007), and fast-paced rhythms than country investors, exacerbating the ***rat race*** in China’s financial and political capitals. City investors in a rapid-developed economy take more risks, act quickly to make money, and experience lower happiness than country investors—following urban and country songbirds. When primed with money, individuals develop self-sufficiency, reduced requests for help, helpfulness to others, intimacy, and social interaction (Vohs et al., 2006).

Country investors share a relaxed, easy-going tempo and green environment. Prosocial behavior mitigates the adverse effects of daily stress (Raposa et al., 2016). Spending time on social interactions, generosity, and emotional support helps consumers weather the stormy stock volatility in a slower, developing economy. Country investors exhibit higher risk aversion and behavioral plasticity (Brockner, 1988), change stock portfolios less often, and are happier than city investors. We theorize that the *combination* of low aspiration, external control, and country domicile leads to high and stable stock happiness amid SHSE Index changes and volatility. Individually, investors take fewer actions than their counterparts.

- Hypothesis 1: The combination of low aspiration, external control, and country domicile leads to the highest and the most stable stock happiness.
- Hypothesis 2: In separate analyses, investors with low aspiration, external control, and country domicile change their stock ratio (stock portfolio) less frequently than their counterparts.

## Method

### Participants

Following IRB approval, we randomly recruited individual private investors from an investment management firm in four regions of China. Investors must be 25–55 years old and have lived in the same domicile for over three years. Investors participated in this field study voluntarily without financial rewards. We assured their confidentiality and obtained their written consent.

### Demographic variables

We obtained investors’ age (median = 36, average = 39.19), gender (male = 110/48.46%, female = 117/51.54%), and stock ratio/portfolio (stock percentages/liquid assets). We classified investors’ monthly salary using eight categories (exchange rate: $1 = ¥6.894) [(0) RMB¥0-¥1,000/$0-$145.05, *n* = 7, (1) ¥1,001-¥2,000/$145.20-$290.11, *n* = 16, (2) ¥2,001-¥4,000/$290.25-$580.21, *n* = 94, (3) ¥4,001-¥8,000/$580.36-$1,160.43, *n* = 69, (4) ¥8,001-¥16,000/$1,160.67-$2,320.86, *n* = 35, (5) ¥16,001-¥32,000/$2,321.00-$4,641.71, *n* = 5, (6) ¥32,001-¥64,000/$4,641.86-$9,283.43, *n* = 0, and (7) ≥ ¥64,001/$9,283.58, *n* = 1]. The investors’ average asset was ¥529,096.64/$76,747.41. The average stock investment was ¥376,831.90/$54,660.85. Only 89 investors (89/227 = 39.21%) changed their portfolios during these 37 days. Regarding domicile, we classified Shanghai (*n* = 60) and Beijing (89) as the city and Shenzhen (45), and Chongqing (33) as the country.

We controlled for gender, age, income, and stock ratio/portfolio changes due to their impacts on decision-making. Women are more risk averse than men (Chen & Tang, 2013; Nelson, 2015). Among MBA students in Shanghai, male investors have higher index happiness and stock happiness than their female counterparts. Age is negatively related to the desire to be Rich. Investors who want to be Rich have a marginally high stock percentage (Tang et al., 2018). The relationships between income and the love of money are negative among highly paid managers (Tang & Chiu, 2003), non-significant among people who change jobs frequently (Tang et al., 2006), and positive among underpaid professors (Luna-Arocas & Tang, 2015). Income and inheritance moderate the relationships between the love-of-money attitudes and short-term and long-term investment decisions (Chaudary et al., 2022). Spanish citizens experienced the dark side of the financial dream (the 30–44 age group, rural residents, and married), whereas others enjoyed the bright side (over-60 age group, unmarried, urban, and 18–29 age group) (Tang et al., 2014).

### Monetary aspiration (level 2)

We quantify investor avaricious monetary aspiration using the 9-item, 3-factor, 5-point Likert-type measure with the following anchors: *strongly disagree* (1), *disagree* (2), *neutral* (3), *agree* (4), and *strongly agree* (5). Scholars have used it in the Chinese context (Tang et al., 2018). We provide one sample item each for Factors Rich: I want to be rich, Motivator: Money is a motivator. Important: Money is important. The Cronbach’s alpha (α) for each factor and the whole scale were 0.81, 0.88, 0.86, and 0.88, respectively.

### Locus of control (level 2)

We used a 29-item forced-choice Internal–External Locus of Control (LOC) measure with six filler items (Rotter, 1966). Each item has two options, for example: (A) Many of the unhappy things in people’s lives are partly due to bad luck, and (B) People’s misfortunes result from the mistakes they make. Option A represents the external locus of control, whereas option B reveals the internal locus of control. We used the scale’s scoring key to calculate each investor’s locus of control score. A high score represents the external locus of control.

### The daily changes of the Shanghai Stock Exchange Index (level 1)

**Fig. 2:**
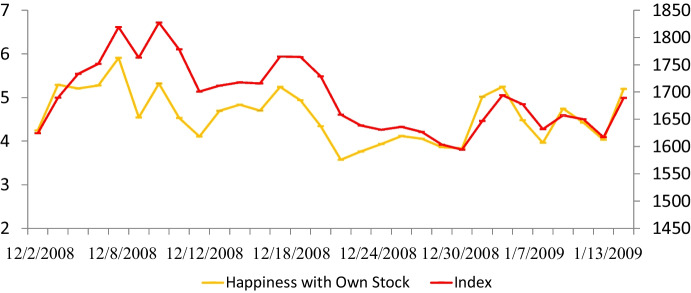
Shanghai Stock Index (SHSE) and Investor Stock Happiness. Note. The X-axis shows the dates of our longitudinal data for the Shanghai Stock Index. The Y-axis on the left indicates stock happiness on a 9-point scale and the one on the right reveals the SHSE

For 37 consecutive trading days, we recorded the objective Shanghai Stock Exchange (SHSE) Index. We calculated the SHSE Index’s ***changes*** for 36 trading days (day_*t*_—day_*t – 1*_, *no comparison* for day_*1*_). The stock index varied from 1594 to 1827 (range 233 points), with the most one-day loss of 77 and the most one-day gain of 72 (Fig. 2). Our data showed “normal” volatility.^4^

### The daily changes of index happiness, stock happiness, and stock portfolio (level 1)

We collected ***subjective*** data by sending text messages to 238 individual investors between 5 and 11 p.m. for 37 consecutive trading days: How happy are you with (1) the Shanghai Stock Exchange Composite Index—Index Happiness and (2) your stocks—Stock Happiness? We used a 9-point scale with *very unhappy* (1), *somewhat unhappy* (3), *neutral* (5), *somewhat happy* (7), and *very happy* (9) as scale anchors. We recorded their ***objective*** daily stock ratio-portfolio changes (stock percentages/liquid assets). Investor portfolio changes may enhance profitability (return on investment, ROI). We deleted 11 individual investors with missing data and retained 227 investors. Overall, investor stock happiness (Y-axis, left scale) mirrored the daily Shanghai Stock Exchange Composite Index (Y-axis, right scale) (Fig. 2).

## Results

### The measurement model of avaricious monetary aspiration

Our confirmatory factor analysis showed an excellent fit between our 3-factor, 9-item aspiration theoretical measurement model and our data (χ^2^ = 48.8558, *df* = 24, *p* = 0.002, GFI = 0.9514, Adjusted GFI (AGFI) = 0.9089, Bentler Comparative Fit Index (CFI) = 0.9773, Bentler-Bonett (NFI) = 0.9567, McDonald Centrality = 0.9433, Bentler-Bonett Non-normed Index = 0.9659, SRMR = 0.0398, and RMSEA = 0.0699). Results offered us confidence in our subsequent analyses.

### Descriptive statistics

Table 1 shows significant variables’ mean, standard deviation, and correlations. Young investors had high avaricious monetary aspirations. Stock happiness was significantly related to investor gender (female), domicile (country), and index happiness, providing preliminary support. Stock happiness was positively associated with an internal locus of control, supporting the literature (Ng et al., 2006), but was unrelated to age, salary, and stock ratio change.

**Table 1:** Means, standard deviation, and correlations among major variables

Variable	Mean	*SD*	1	2	3	4	5	6	7	8
1. Age	39.1850	11.1392								
2. Gender	0.5200	0.5007	0.0407							
3. Salary	2.5683	1.0511	-0.1311*	-0.0767						
4. Domicile	0.3436	0.4760	-0.1447*	-0.0008	-0.0648					
5. Stock Ratio Change	-0.0220	6.1776	0.0110	0.0037	-0.0287	-0.0576				
6. Index Happiness	5.8855	1.6763	-0.0640	-0.0821	0.1074	0.0606	-0.0002			
7. Aspiration	3.7112	0.7604	-0.1543*	0.0435	0.0651	0.0180	0.0860	-0.0118		
8. Locus of Control	10.5771	3.8244	0.0331	0.1561*	-0.0016	-0.0681	0.1373*	-0.0745	0.0910	
9. Stock Happiness	5.2996	1.7544	-0.0549	-0.2128**	0.0560	0.1729**	0.0067	0.5278***	-0.0504	-0.1367*

*N* = 227

Gender: 0, female; 1, male

Salary: (0) ¥0 ‒ ¥1,000 (1) ¥1,001 ‒ ¥2,000 (2) ¥2,001 ‒ ¥4,000 (3) ¥4,001 ‒ ¥8,000 (4) ¥8,001 ‒ ¥16,000 (5) ¥16,001 ‒ ¥32,000 (6) ¥32,001 ‒ ¥64,000 (7) ≥ ¥64,001

Domicile: 0, city; 1, country

^*^p < .05, **p < .01, ***p < .001

### Part I—cross-level analysis

Researchers achieve *higher* power (1 – β) by employing *larger* samples at Level 2 rather than Level 1 (Aguinis et al., 2013). We had 227 investors at Level 2 and 36 repeated measures (changes) at Level 1. Our ratio (227/36) exceeded the 30/30 requirement for cross-level analysis. Multilevel modeling conceptualizes the investors as a random sample from a larger population of investors (a random factor). Model 1 is an unconditional model without predictors to access between-persons variation in stock happiness in our stepwise modeling. Model 2 adds the level-1 predictor (the fixed effect) to obtain the average stock happiness change. In Model 3, to understand if the average difference in stock happiness varies across individual investors, we add a level-1 predictor’s random effect. Model 4 adds the level-2 predictors to the previously estimated random intercept and slope model. Tables 2, 3, 4, and 5 present our step-by-step multilevel analysis results.

**Table 2:** Results of longitudinal cross-level analysis on stock happiness

	Model 1	Model 2	Model 3	Model 4
	Estimate	Estimate	Estimate	Estimate
Fixed Effect				
Intercept	4.9145***	4.8746***	4.8711***	5.1496***
Index Change		0.0113***	0.0113***	0.0008
Index Happiness				1.4573***
Aspiration				-0.1586
Locus of Control				0.0085
Domicile				-0.0006
Index Change * Index Happiness				-0.0014
Index Change * Aspiration				-0.0001
Index Change * Locus of Control				-0.0008
Index Change * Domicile				0.000004
Index Happiness * Domicile				-0.0013***
Aspiration * Locus of Control				0.0754
Aspiration * Domicile				0.0002
Locus of Control * Domicile				-0.0001
Index Change * Index Happiness * Aspiration				-0.0016
Index Change * Index Happiness * Locus of Control				0.0006*
Index Change * Index Happiness * Domicile				0.000001
Index Change * Aspiration * Locus of Control				0.0027***
Index Change * Aspiration * Domicile				0.0000001
Index Change * Locus of Control * Domicile				0.000001***
Index Happiness * Aspiration * Locus of Control				-0.1389***
Index Happiness * Aspiration * Domicile				0.00001
Index Happiness * Locus of Control * Domicile				0.00004***
Aspiration * Locus of Control * Domicile				-0.0002
Index Change * Index Happiness * Aspiration * Locus of Control				0.0003
Index Change * Index Happiness * Aspiration * Domicile				0.000004**
Index Change * Index Happiness * Locus of Control * Domicile				-0.000001***
Index Change * Aspiration * Locus of Control * Domicile				-0.000004***
Index Happiness * Aspiration * Locus of Control * Domicile				0.0002***
Index Change * Index Happiness * Aspiration * Locus of Control * Domicile				-0.0000003***
Age	-0.0054	-0.0054	-0.0051	-0.0030
Gender	-0.3565*	-0.3566*	-0.3528*	-0.2849
Salary	0.0424	0.0424	0.0397	0.0690
Stock Ratio Change	0.0106**	0.0117***	0.0124***	0.0109***
Error Variance				
Level-1	1.2518***	1.0551***	0.9007***	0.4813***
Level-2 Intercept	1.3455***	1.3510***	1.3629***	1.2651***
Index Change			0.0001***	0.00002***
Model Fit				
AIC	26,673.8	25,275.6	24,438.5	18,421.1
BIC	26,698.1	25,303.3	24,469.7	18,547.5

Model 1: ICC = 1.3455/(1.3455 + 1.2518) = 51.80%. Results supported our cross-level analyses

^*^p < .05, **p < .01, ***p < .001

**Table 3:** Comparison between city and country

	Model 5 City			Model 6 Country			City vs. Country
	Estimate	Standard Error	*t*-Value	Estimate	Standard Error	*t*-Value	*F*-Value
Fixed Effect							
Intercept	4.2145	0.5210	8.09***	5.5082	0.4893	11.26***	
Index Change	0.0091	0.0008	11.00***	0.0158	0.0013	12.44***	
Aspiration	0.0163	0.1419	0.11	-0.0807	0.1359	-0.59	
Locus of Control	-0.0763	0.0265	-2.88**	-0.0265	0.0312	-0.85	
Index Change * Aspiration	0.0002	0.0011	0.20	0.0009	0.0017	0.57	
Index Change * Locus of Control	0.0003	0.0002	1.26	0.0002	0.0004	0.60	
Aspiration * Locus of Control	-0.0417	0.0363	-1.15	-0.0031	0.0402	-0.08	
Index Change * Aspiration * Locus of Control	-0.0002	0.0003	-0.70	0.0001	0.0005	0.12	3.86*
Age	0.0056	0.0095	0.59	-0.0177	0.0101	-1.76	
Gender	-0.4041	0.2095	-1.93	-0.0578	0.2170	-0.27	
Salary	0.1015	0.1066	0.95	-0.0108	0.0877	-0.12	
Stock Ratio Change	0.0119	0.0043	2.80**	0.0075	0.0048	1.54	
Error Variance							
Level-1	0.8681	0.0173	50.16***	0.8954	0.0248	36.18***	
Level-2 Intercept	1.4801	0.1749	8.46***	0.7509	0.1251	6.00***	
Index Change	0.0001	< .0001	7.22***	0.0001	< .0001	5.35***	

^*^p < .05, **p < .01, ***p < .001

**Table 4:** Comparison between city and country for high aspiration investors

	High Aspiration Investor						
	Model 7 City			Model 8 Country			City vs. Country
	Estimate	Standard Error	*t*-Value	Estimate	Standard Error	*t*-Value	*F*-Value
Fixed Effect							
Intercept	1.9927	1.4241	1.40	5.4541	1.1659	4.68**	
Index Change	0.0085	0.0018	4.70***	0.0149	0.0037	4.03**	11.10***
Locus of Control	-0.1140	0.0567	-2.01*	-0.0858	0.0450	-1.90	5.06*
Index Change * Locus of Control	0.0001	0.0004	0.27	0.0001	0.0008	0.08	0.09
Age	0.0162	0.0240	0.67	-0.0143	0.0205	-0.70	
Gender	-0.0593	0.4719	-0.13	-0.2806	0.6326	-0.44	
Salary	0.7298	0.3060	2.39*	0.1463	0.1832	0.80	
Stock Ratio Change	0.0217	0.0097	2.24*	0.0023	0.0126	0.19	
Error Variance							
Level-1	1.0415	0.0469	22.20***	1.0527	0.0737	14.28***	
Level-2 Intercept	1.4290	0.3829	3.73***	0.2979	0.1336	2.23**	
Index Change	0.0001	< .0001	3.02**	0.0001	< .0001	2.12**	

^*^p < .05, **p < .01, ***p < .001

**Table 5:** Comparison between city and country for low aspiration investors

	Low Aspiration Investor						
	Model 9 City			Model 10 Country			City vs. Country
	Estimate	Standard Error	*t*-Value	Estimate	Standard Error	*t*-Value	*F*-Value
Fixed Effect							
Intercept	5.6997	0.7605	7.49***	4.5596	0.9374	4.86***	
Index Change	0.0104	0.0021	4.99***	0.0116	0.0027	4.37***	1.01
Locus of Control	-0.0570	0.0405	-1.41	0.0156	0.0890	0.18	16.18***
Index Change * Locus of Control	0.0003	0.0006	0.54	-0.0010	0.0009	-1.08	3.95*
Age	-0.0147	0.0120	-1.23	-0.0107	0.0208	-0.52	
Gender	0.0757	0.3168	0.24	1.2163	0.5083	2.39*	
Salary	-0.1983	0.1750	-1.13	0.1056	0.1631	0.65	
Stock Ratio Change	0.0206	0.0123	1.68	0.0075	0.0072	1.04	
Error Variance							
Level-1	1.1345	0.0530	21.42***	0.6385	0.0400	15.97***	
Level-2 Intercept	0.5893	0.1690	3.49***	0.7973	0.2977	2.68**	
Index Change	0.0001	< .0001	3.02**	0.0001	< .0001	2.41**	

^*^p < .05, **p < .01, ***p < .001

In Model 1 (Table 2), we controlled for four variables (age, gender, salary, and stock ratio/portfolio change), helping us enhance the internal validity and eliminate confounding factors. Females and investors with stock portfolio changes showed significantly higher stock happiness than their counterparts. However, age and income-salary were unrelated to stock happiness. We calculated the intraclass correlation (ICC) using controlled variables. ICC explained 51.80% of the variance in stock happiness across investors, supporting our multilevel analysis (Table 2, ICC = 51.80% = 1.3455/(1.3455 + 1.2518)).

Figure 1 shows that stock index *changes* impact stock happiness directly and indirectly through index happiness. Since investors’ index happiness reflects their *subjective* reactions, we focus on the ***objective*** stock index changes. We exclusively investigated the significant cross-level impacts of aspiration, control, domicile (Level 2), and daily stock index change (Level 1) on investors’ daily stock happiness (Level 1) (Fig. 1, Path 1).

## Three-dimensional visualization

**Fig. 3:**
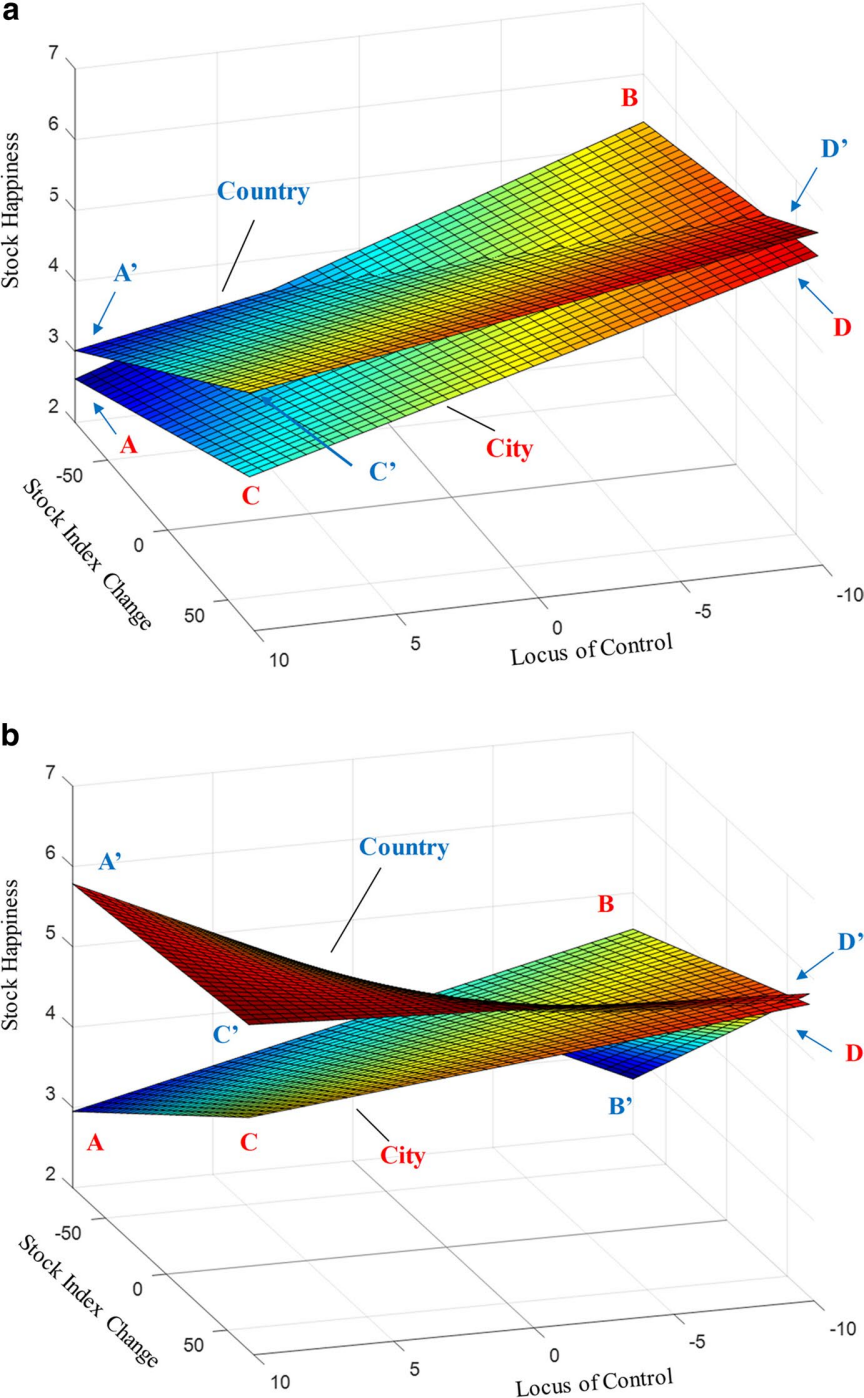
Cross-level longitudinal investor stock happiness as a function of the longitudinal stock index changes (X-axis), locus of control (Y-axis), investor domicile (city vs. country), and avaricious monetary aspiration, panels **A** (high aspiration) and **B** (low aspiration). Panel **A**: High Aspiration Investors. The difference in slopes between the city and country is non-significant. Country investors have higher stock happiness than city investors, except for internal country investors with the most substantial losses. With internal locus of control, city (6.288) and country investors (6.614) have the highest stock happiness when experiencing the most substantial stock index increases. With an external locus of control, city (2.615) and country investors (3.116) have the lowest stock happiness when experiencing the most considerable stock index declines. Internal city investors display *the smallest* increase in stock happiness (.964, from 5.324 to 6.288). External city investors show a moderate increase (1.474, from 2.615 to 4.089), whereas internal country investors demonstrate the second most significant increase in stock happiness (2.058, from 4.556 to 6.614). External country investors illustrate *the largest* fluctuation of stock happiness (2.160, from 3.116 to 5.276). Panel **B**: Low Aspiration Investors. The difference in the slopes between the city and the country is significant. Internal city investors exhibit a moderate increase in stock happiness (1.087, from 4.549 to 5.636). External city investors create the second most considerable oscillation (1.948, from 2.951 to 4.899). Internal country investors produce *the most significant* fluctuation (3.084, from 2.683 to 5.767). External country investors reveal *the most negligible* changes (0.272, from 5.783 to 6.055). The SHSE Index varied from 1594 to 1827 (range: 233 points), with the most one-day loss of 77 and the most one-day gain of 72. The reference point, 0, indicated no change in the SHSE Index

Hypothesis 1 investigates the significant cross-level four-way interaction effect on stock happiness (Table 2, Model 4, *p* < 0.001). We used MatLab to plot our 3-D surfaces of stock happiness. Figure 3 panels A and B show investors with high and low aspirations, respectively. Each panel has two surfaces (city vs. country).

For the stock index change (X-axis), the reference point (zero) suggests no difference between day_t_ and day_t-1_, whereas negative values signal ***losses*** and positive scores indicate ***gains***, reflecting index volatility. The Y-axis shows that the investor locus of control has a neutral point (zero), positive values (external control), and negative values (internal control). The Z-axis demonstrates changes in daily stock happiness across 36 days. We classified investors into high and low aspirations using a formula (µ ± σ). Table 3 illustrates the significant differences in the three-way interaction effect (Index change*Aspiration*Locus of Control) between the city (Model 5) and country (Model 6) investors (*F* = 3.86, *p* < 0.05).

### Panel A: high avaricious aspiration investors

The country investors had higher overall stock happiness than the city investors, except for internal investors in the loss domain. The difference in the ***slope*** between the city and the country (index change*locus of control interaction) was non-significant (*F* = 0.09, *p* > 0.05, Table 4, Model 7 vs. Model 8). With an internal locus of control, city and country investors had great stock happiness when they had the most substantial gains, supporting the general expectations. With an external locus of control, city and country investors had low stock happiness when they had the most significant losses. Country investors with *external* control illustrated the most substantial stock happiness increases in the boom-and-bust cycles (2.160, from 3.116 to 5.276). City investors with internal control displayed minuscule stock happiness changes (0.964, from 5.324 to 6.288). More city investors changed their portfolios (41.37%) than country investors (33.33%) among high-aspiration investors. The difference in stock ratio/portfolio changes between the two was 8.04%.

### Panel B: low avaricious aspiration investors

Panels B and A are similar. However, the difference in the ***slope*** between the two surfaces (city vs. country) was significant (*F* = 3.95, *p* < 0.05, Table 5, Model 9 vs. Model 10). Amid stock volatility changes across 36 trading days, investors with ***low aspiration***, an ***external control***, and a ***country domicile*** showed the ***highest*** level and the most ***stable*** longitudinal stock happiness—with the lowest stock happiness changes-fluctuations (0.272, from the lowest-5.783 to the highest-6.055). However, those with low aspirations, an external control, and city domicile illustrated the second-highest stock happiness increase (1.948, from 2.951 to 4.899). Investors with internal control and country domicile established the highest stock happiness enhancement (3.084, from 2.683 to 5.767). Investors with internal control and city domicile showed a modest improvement.

Overall, the country investors revealed the highest behavioral plasticity level—the internal investors have the most significant changes and are the most vulnerable. The external investors, on the other hand, had the lowest. Compared with high-aspiration investors (city: 41.37% vs. country: 33.33%), low-aspiration investors showed lower stock ratio changes (city: 25.93% vs. country: 26.67%). The difference between the two was negligible, 0.74%.

Regardless of volatility, investors with low aspirations, *external* control, and a country domicile show the highest and the most stable, sustainable stock happiness, revealing the *most negligible* stock happiness changes, supporting Hypothesis 1. However, investors with low aspirations, *internal* control, and a country domicile have the highest stock happiness fluctuation.

### Part II—investor behaviors

We analyzed investors’ actual stock ratio changes across three independent variables separately. Our crosstabulation of aspiration for money (high vs. low) and the change of the stock ratio (change vs. no change) showed the chi-square was not significant (*χ*^*2*^ (1) = 2.2325, *p* = 0.1351). The odds ratio and relative risk analysis result indicated that low-aspiration investors were ***1.64*** times less likely to change the stock ratio than high-aspiration investors (Table 6). Similarly, external investors were ***1.35*** times less likely to change the portfolio than their internal counterparts (*χ*^*2*^ (1) = 0.8901, *p* = 0.3455) (Table 7). The country investors were ***1.15*** times less likely to change their portfolio than their city counterparts (*χ*^*2*^ (1) = 0.1534, *p* = 0.6953) (Table 8). Despite non-significant findings, investors with low aspiration, external control, and a country domicile were 1.64 times, 1.35 times, and 1.15 times less likely to make stock portfolio changes than their counterparts.

**Table 6:** Crosstabulation of aspiration for money and change of stock ratio

Stock ratio change				
Aspiration		Change	No change	Total
High	Frequency	16	25	41
	Percent	19.28	30.12	49.40
	Row %	39.02	60.98	
	Column %	61.54	43.86	
Low	Frequency	10	32	42
	Percent	12.05	38.55	50.60
	Row %	23.81	76.19	
	Column %	38.46	56.14	
Total	Frequency	26	57	83
	Percent	31.33	68.67	100

Chi-square results: *χ*^*2*^ (1) = 2.2325, *p* = .1351, Likelihood ratio *χ*^*2*^ (1) = 2.2469, *p* = .1339, Continuity Adj. *χ*^*2*^ (1) = 1.5813, *p* = .2086, Mantel–Haenszel *χ*^*2*^ (1) = 2.2056, *p* = .1375, Phi coefficient = .1640, Contingency coefficient = .1618. Odds ratio and relative risks: Odds ratio = 2.0480, 95% confidence limits [0.7940 5.2828], Relative risk (Column 1) = 1.6390 [0.8449 3.1795], Relative risk (Column 2) = .8003 [.5943 1.0777]. The high aspiration investors were **1.6390** times more likely to change the stock ratio than the low aspiration investors

**Table 7:** Crosstabulation of locus of control and change of stock ratio

Stock ratio change				
Locus of control		Change	No change	Total
Internal	Frequency	16	27	43
	Percent	19.28	32.53	51.81
	Row %	37.21	62.79	
	Column %	59.26	48.21	
External	Frequency	11	29	40
	Percent	13.25	34.94	48.19
	Row %	27.50	72.50	
	Column %	40.74	51.79	
Total	Frequency	27	56	83
	Percent	32.53	67.47	100

Chi-square results: *χ*^*2*^ (1) = 0.8901, *p* = .3455, Likelihood ratio *χ*^*2*^ (1) = 0.8943, *p* = .3443, Continuity Adj. *χ*^*2*^ (1) = 0.5027, *p* = .4783, Mantel–Haenszel *χ*^*2*^ (1) = 0.8794, *p* = .3484, Phi coefficient = .1036, Contingency coefficient = .1030. Odds ratio and relative risks: Odds ratio = 1.5623, 95% confidence limits [0.6167 3.9578], Relative risk (Column 1) = 1.3531 [0.7166 2.5547], Relative risk (Column 2) = .8661 [.6423 1.1679]. The internal-locus-of-control-investors were **1.3531** times more likely to change the stock ratio than the external-locus-of-control-investors

**Table 8:** Crosstabulation of investor domicile and change of the stock ratio

Stock ratio change				
Investor domicile		Change	No change	Total
City	Frequency	19	37	56
	Percent	22.89	44.58	67.47
	Row %	33.93	66.07	
	Column %	70.37	66.07	
Country	Frequency	8	19	27
	Percent	9.64	22.89	32.53
	Row %	29.63	70.37	
	Column %	29.63	33.93	
Total	Frequency	27	56	83
	Percent	32.53	67.47	100

Chi-square results: *χ*^*2*^ (1) = 0.1534, *p* = .6953, Likelihood ratio *χ*^*2*^ (1) = 0.1547, *p* = .6941, Continuity Adj. *χ*^*2*^ (1) = 0.0200, *p* = .8874, Mantel–Haenszel *χ*^*2*^ (1) = 0.1515, *p* = .6971, Phi coefficient = .0430, Contingency coefficient = .0429. Odds ratio and relative risks: Odds ratio = 1.2196, 95% confidence limits [0.4513 3.2959], Relative risk (Column 1) = 1.1451 [0.5763 2.2754], Relative risk (Column 2) = 0.9389 [.6897 1.2781]. The city-investors were **1.1451** times more likely to change the stock ratio than the country-investors

## Discussion

### Theoretical implications

We answer Nobel Laureate Daniel Kahneman’s call (2011, p. 298), incorporate “attitude toward money,” and investigate Chinese investors’ stock happiness and portfolio changes. Most famous for his portfolio theory, asset pricing, and the efficient-market hypothesis, Fama (1998) stressed the difficulties in predicting stock price movements in the short term. Shiller (2015), however, predicted the 2008 housing crash in the long run. Since individual private investors have very little control over stock volatility, we also included investors’ locus of control in our study. Individuals with an internal locus of control tend to have high satisfaction and well-being (Spector et al., 2002). However, high internal locus-of-control investors’ attempt to control the uncontrollable stock volatility may cause dire “negative attitudinal or behavioral outcomes” (Ng et al., 2006, p. 1,074). The importance of contextualization (Johns, 2017) and place identity motivates us to incorporate investor domicile (city vs. country) in four regions of China. We theorize that investors’ avaricious monetary aspiration, locus of control, and domicile (Level 2 variables) help them frame the critical concerns—the longitudinal SHSE volatility (Level 1 variables) and demonstrate their sustainable daily subjective (stock happiness) and objective (portfolio changes). To the best of our knowledge, scholars have never done this before.

In this study, we ask the following question: Who are the investors with the highest and most sustainable stock happiness, and why? First, our correlational data offer some simple observations. Interestingly, young investors have higher avaricious monetary aspirations than their older counterparts, supporting the literature (Tang et al., 2018a, b). As expected, females (gender), country investors (domicile), and investors with high index happiness demonstrate increased stock happiness. Stock happiness was positively associated with an internal locus of control. This finding supports most people’s expectations and the literature (Ng et al., 2006). Investors with an internal locus of control tend to take action, reap their rewards, and enjoy their ROI. However, stock happiness is unrelated to age, salary, and stock portfolio changes.

Second, our cross-level discoveries provide additional clues. Prospect theory frames decision-making under uncertainty in the gains-losses domains and high-low probability. In the present study, the SHSE varied from 1594 to 1827 (range 233 points), with the most one-day loss of 77 and the most one-day gain of 72 (Fig. 2). Our stock index changes reflected “normal” volatility. Therefore, our findings of investors’ stock happiness and portfolio changes apply to individual private investors’ reactions to the ordinary boom-and-bust cycles and ordinary citizens. Our 227 private investors with real stock investments ($54,660.85) differ slightly from investors who were MBA students. In the present study, women have higher stock happiness than men. For the MBA sample, males show significantly higher index happiness and stock happiness than females (Tang et al., 2018). This notion deserves future scholars’ theory development and testing. We list our novel discoveries below:

Our major contribution to the behavioral economics literature is that the combination of low aspiration, external control, and country domicile robustly ***predicts*** investors’ highest and most sustainable stock happiness with minimum fluctuation amid the boom-and-bust cycles for 36 consecutive trading days. Independently, investors with low aspirations, external control, and a country domicile are 1.64 times, 1.35 times, and 1.15 times less likely to make portfolio changes than their counterparts. Interestingly, ***aspiration*** is slightly more potent than the locus of control and their domicile. Following Mark Twain, “action speaks louder than words,” behaviorally, less is more. Avoiding avaricious risky-and-panicky decision-making reduces stress and enhances investors’ stable and robust sustainable stock happiness.

This study is *not* a “one-shot” game with “nothing at stake” (Thaler, 2015) and makes vital contributions to prospect theory, behavioral economics, stress management, health, and well-being. Our empirical data support Kahneman’s advice: “Closely following daily fluctuations is a losing proposition because the pain of the frequent small losses exceeds the pleasure of the equally frequent small gains” (2011, p. 339).

Reacting quickly to the SHSE Index volatility like the executive monkeys (Brady, 1958) is *not* beneficial to consumers’ health. To achieve true happiness, private investors, and ordinary citizens, in general, must become the master of money and treat money as a tool, not a drug (Lea & Webley, 2006). We encourage people to move beyond happiness and focus on a meaningful life (Baumeister et al., 2013). Research shows that “The love of money is the root of all evils” (Tang & Chiu, 2003; Tang et al., 2018b, 2022). We must avoid serving mammon which may lead to dire consequences and becoming slaves to money. Investors must focus on their long-term investment value rather than leveraging the daily index fluctuations, maximizing expected utility. The former helps investors deliberately evade stock volatility and improves their daily emotional quality of life and serenity. Following Merton’s (1968) Matthew Effect, we propose ***the Matthew Effect in monetary wisdom***. The rich—with monetary wisdom—joy, peace, and serendipity—get richer—financially, physically, and psychologically, achieving ultimate serenity (Judge & Hurst, 2008).

### Avaricious aspiration

Following the clashes of sacred (self-transcendence) and secular values (self-enhancement) (Grouzet et al., 2005; Schwartz, 1992), scholars have moved beyond materialistic values and found religions and sacred values for possible relief. *Mindfulness* training—Mindfulness-Based Stress-Reduction (MBSR)—rooted in Buddhism, reduces dishonesty directly and indirectly via lower aspirations (Gentina et al., 2021; see Burton et al., 2016). A robust difference exists between those with and those without MBSR training. Mindfulness prevails among those who completed the training within one year and practiced it within two years. Reducing investors’ greed helps them achieve ultimate serenity.

Based on the 2013 Chinese General Social Survey (CGSS, the Renmin University of China, Beijing) involving 10,016 households across 31 provinces of China, believers in all religions inspire more sustainable HOPE (Help Ourselves Protect the Environment) than atheists (Mo et al., 2022). Taoism and Buddhism believers have higher HOPE than other faiths. Believers practicing institutionalized rituals in organized religions display higher HOPE than those without formalized worship. When researchers yoke intrinsic religiosity (God) and love of money (mammon) in a formative theoretical SEM model, surprisingly, males reduce dishonesty by omission; females enhance honesty by commission (Chen et al., 2022b). A wandering mind is unhappy (Killingsworth & Gilbert, 2010). Mindfulness-Based Stress Reduction training, Eastern (Taoism, Buddhism), and Western religions (Christianity) may help consumers increase their awareness of the present and curb materialistic and hedonistic values, reducing stress and achieving happiness (Gentina et al., 2021; Tang & Tang, 2010; Zhou et al., 2018). Future researchers may empirically explore these issues in other contexts, regions, religions, and cultures.

### External-internal locus of control

Non-avaricious, ***internal control***, and country investors have the ***highest*** stock happiness’ ***fluctuation***, becoming the ***most vulnerable*** investors in our study. However, non-avaricious, ***external*** control, and country investors demonstrate the highest and the most stable longitudinal stock happiness. Relinquishing their control and making fewer portfolio changes contribute to their long-lasting and happy feelings amid the *uncontrollable* Index volatility (Ng et al., 2006).

### Domicile: country vs. city

The longitudinal Shanghai Stock Exchange Composite Index impacted investor stock happiness and behaviors in all four regions of China, not limited to Shanghai. The omnibus environment powerfully molds our thoughts, feelings, and actions across time and space (Oishi, 2015). We classified Shanghai and Beijing as the city and Shenzhen and Chongqing as the country using objective and subjective criteria. Our 3-D visualization reveals robust differences between investors in the city and country, supporting our classification of these four regions, the inclusion of identity (Akerlof & Kranton, 2000), the importance of environmental capital, and the novel presentation of investor stock happiness. Our novel findings related to investors’ domiciles make a vital contribution to the literature.

Below is an exciting story on nudge and *choice architecture* (Thaler, 2015). In Chinese history, Mencius’ mother raised her son alone. To improve her son’s physical environment, she moved from a funeral home and cemetery neighborhood to a slaughterhouse and, finally, to a school. These three moves inspired her son, Mencius, to imitate and become a famous scholar due to the mere exposure to a conducive and stimulating academic environment. Chinese people consider Mencius (372–289 BC) the second Sage, only after Confucius (551–479 BC). The environment serves as an *engine* for success or a *brake* on their ambitions (Chetty et al., 2014). Our visualization supports our Monetary Wisdom, helping individual private investors and ordinary citizens make healthy, happy, and wealthy decisions and save lives, supporting behavioral economics (Kahneman, 2011; Thaler, 2015). This notion deserves future scholars’ further empirical investigation and theory development and testing.

## Practical implications

How can we improve the choice architecture and nudge investors to be happy (Hauser et al., 2018; Thaler, 2015)? At the individual level, we must budge their minds by *understanding* their psychological-subconscious beliefs about aspiration, control, and domicile, *removing* barriers, and *providing* a conducive environmental context. Moreover, comparing investors’ values (aspiration and control) with those of the happiest investors helps them visualize the potential gaps between “where they are” and “where they need to be.” Further, visualization of their SMART (specific, measurable, ambitious, realistic, and time-bound) goals (Latham et al., 2010) helps individuals achieve their goals (Habakkuk 2:2; Cheema & Bagchi, 2011; Howard et al., 2015). We must focus on what we have and be grateful for our possessions. Let our lives be free from love of money but be content with what we have (Hebrews 13:5). Comparing themselves with the poor, showing gratitude, and reflecting on what they have abundantly received may nudge them toward improving their holistic decision-making. The urban and country songbirds and Aesop’s fable of the *city* mouse and *country* mouse remind us that we must become ***choice architects***, make wise choices, take action, and embrace the ***environmental capital***. Stay away from the stressful milieus and the rat race. As mentioned, the environment serves as an *engine* for success or a *brake* on their ambitions (Chetty et al., 2014). Follow Mencius’ mother as a role model, who moved her home three times, inspiring Mencius to become the second Sage, domicile matters.

At the organizational level, talent management strategy (training and development) reduces burnout and enhances job and life satisfaction and the sales commission (Srivastava & Tang, 2022). Interestingly, life satisfaction (not job satisfaction) mediates the relationships between talent management strategy and the sales commission. Scholars and practitioners must expand their vision, adopt a new lens, and holistically frame their attention to the whole of individuals. At the global level, MNEs must develop fair compensation systems across different parts of the world to increase pay satisfaction and justice perceptions and curb people’s greedy desires. MNEs’ (un)ethical values and avaricious monetary aspirations (love of money) at the top organizational echelon create a trickle-down (cascade) effect on lower-level employees at the individual, organization, and global levels (Al Halbusi et al., 2022; Tang, 2021). This powerful social norm in the environmental context impacts individuals, which deserves researchers’ and managers’ future empirical attention (Tang et al., 2018b, 2022). High income reduces greedy aspirations (Tang & Chiu, 2003). Wisdom contributes to health and happiness, refuting the folk belief that “Ignorance is bliss” (Judge et al., 2010, p. 463).

Many factors contribute to the selection of homes (socio-economic status, demographic variables, job opportunities, personality, quality of life, future aspirations, and locations). Investors and ordinary citizens who live in the city must avoid high exposure to money, the SHSE Index, time pressure (time is money) (DeVoe & Pfeffer, 2007), and fast-paced rhythms (Lyles, 2015). City investors in a rapid-developed economy must reduce their risk-taking investment, expand their horizons, and change short-term decisions to long-term decisions to reduce stress and time pressure and increase their happiness. People need to belong (Baumeister & Leary, 1995). Prosocial behaviors (caring about others, becoming helpful, enhancing intimacy and social interaction) mitigate the adverse effects of daily stress (Preston & de Waal, 2002; Raposa et al., 2016).^5^ Helping others, spending money on others, and donating money, time, and expertise to charity, church, and communities will enhance our happiness and create meaning in our lives. Become a giver (not a taker) and live a meaningful life (Baumeister et al., 2013). God loves a cheerful giver (2 Corinthians 9:7). Developing a hardy personality (control, challenge, and commitment) helps people combat stress (Tang & Hammontree, 1992). Social identity impacts individual private investors’ longitudinal subjective happiness and objective portfolio changes. Our novel discoveries add a new twist to the existing economic literature, supporting Nobel Laureate Akerlof’s notion of *social identity* and the importance of *contextualization* in empirical studies (Johns, 2017; Rousseau & Fried, 2001). When investors step back and relax, they may not only enjoy peace but also calm the stormy financial turbulences. Following the prospect theory, the pain of the frequent small losses exceeds the pleasure of the equally frequent small gains (Kahneman, 2011, p. 339).

As an alternative to changing our domiciles and moving to rural areas, 20 to 30 min of *nature experiences* have the most significant impact (Hunter et al., 2019; Wicks et al., 2022). Playfulness promotes well-being. Taking a *nature pill* reduces anxiety, attracts synergy, and improves happiness. We nudge people to take periodic *vacations* in natural locations, listen to songbirds, smell the roses to renew a relaxed spirit, expose ourselves to nature, green scenery, tranquility, a serendipitous country surrounded by concentio (people or songbirds singing in harmony), freedom, and peace (De Bloom et al., 2011).

Teresa Amabile, Harvard Business Professor, stated the following: When creativity is under the gun, it usually ends up getting killed. In Sweden, Fika coffee breaks are legally protected and mandatory in many firms. Fika breaks allow employees to relax, slow down, and leave work behind in a social setting, creating a conducive work environment, fortifying the least stressed workforce worldwide, reducing accidents, and enhancing creativity, happiness, and productivity.

Globally, empirical studies showed that satiation occurred at $75,000 (Kahneman & Deaton, 2010) or $95,000 for life evaluation and $60,000 to $70,000 for emotional well-being (Jebb et al., 2018), which may vary across cultures. Chinese investors have an average income lower than these global satiation points, helping us explain Chinese people’s risk-taking actions in the stock markets. It is essential to let our life be free from the love of money but be content with what we have. We utilize a Ulysses contract to silence irrational thinking, helping us become healthier, happier, and wealthier than before.

Spending money on others enhances happiness. Reminders of mortality lead to giving to others (Dunn et al., 2008, 2020). Billionaires such as Warren Buffet, Priscilla Chan, and Mark Zuckerberg pledged to share their wealth through philanthropy. Giving wealth away creates happiness and meaning and stores the treasures in many people’s hearts. As of March 27, 2022, the COVID-19 pandemic has caused 480 million cases and 6.145 million deaths worldwide. “There is a realm of time where the goal is not to have but to be” (Heschel, 1951, p. 3). 

Daniel Kahneman (2022) stated “Happiness is a meaningful and elusive quality in every person’s life” (p. 5). He encouraged us to think about two different “types of happiness—being happy in your life, and being happy about your life” (p. 5). The former (being happy in your life) is related to the momentary experience and subjective good and pleasant feelings—experienced happiness. People are the happiest when they spend time with people they love and who love them. The latter (being happy about your life) focuses on life satisfaction. Life satisfaction reflects our general satisfaction from life regarding our objective success and achievements. In this study, our exploration of monetary wisdom simultaneously deals with expected utility (objective success and achievements) and ultimate serenity (subjective happiness). Our 3-D visualization provides a brand-new perspective, capturing the essential spirit of the prospect theory and revealing a substantial and exemplary demonstration of behavioral economics. We further expand the notion and nuge people to turn greed into gratitude, live the idyllic present with passion, accept serendipity in country living, and achieve both types of happiness. Our cross-disciplinary implications help ordinary citizens and consumers make happy, healthy, and wealthy decisions, saving lives, including our own.

## Limitations and future research

Our data did not reflect volatility at the peak of the financial crisis. Due to our longitudinal study, our sample size was small. Future scholars may include additional psychological and environmental constructs, objective return on investment, ultimate financial performance, large sample, long duration, explore a different Stock Index in various countries, compare investors across cultures in developed and emerging markets, and conduct laboratory experiments to verify our present findings. Our innovative discoveries apply to financial investors and ordinary citizens.

## Conclusion

Our discoveries based on 227 investors’ longitudinal data for 36 consecutive trading days across four regions of China suggest: The combination of low aspiration, external control, and country domicile leads to the highest, longitudinal, and sustainable happiness with minor fluctuation. In three separate analyses, investors with lower aspirations, external control, and country domicile tend to exhibit fewer portfolio changes than their counterparts. Behaviorally, less is more, demonstrating consistency between subjective feelings and objective stock actions.

Monetary wisdom asserts: Individuals apply deep-rooted values to frame their critical concerns in the immediate and omnibus contexts to maximize their expected utility and ultimate serenity across people, context, and time at the individual, organization-industry, and country-global levels. Our longitudinal study expands prospect theory, makes robust contributions to behavioral economics, and nudges investors and ordinary citizens toward wiser financial decisions, healthier lives, and greater happiness. Our powerful polemic will elegantly stimulate future theoretical advancement, empirical refinement, potential philosophical hermeneutics, and the betterment of both science and practice of stock investment, stress management, business ethics, and psychological well-being. The life you save may be your own.

We presented portions of this paper at the International Convention of Psychological Science, Paris, France, March 7–9, 2019.

Submission declaration and verification: We have not published this article before.

## Data Availability

(https://dataverse.harvard.edu/dataverse/Ningyu). (https://dataverse.harvard.edu/dataset.xhtml?persistentId=doi:10.7910/DVN/NDMTIX) Hai Fu Tong Dataset.

## Footnotes

1 We thank Editor-in-Chief Professor Allan K. K. Chan and two anonymous reviewers for their valuable feedback, constructive suggestions, and insightful comments.

2 Here are some examples: the Czech Republic, Kazakhstan, Kyrgyzstan, Macedonia, Pakistan, Poland, Sri Lanka, Swaziland, Uganda, Uzbekistan, Vietnam, and Zimbabwe (Tang, 2021).

3 NPR: Hidden Brain: < Zipcode destiny: The persistent power of place and education (December 9, 2019). https://www.npr.org/transcripts/786469762

4 During the financial crisis, SHSE Index had a very significant 4,385.36-point drop. Our data indicated normal index volatility (between December 2, 2008, and January 13, 2009).

5 How to make stress your friend (Kelly McGonigal). https://www.youtube.com/watch?v=RcGyVTAoXEU&t=543s
